# Adsorption-Induced Pore Volume Deformation: Implications for Excess Adsorption in Kerogen Matrices

**DOI:** 10.1021/acs.langmuir.6c00872

**Published:** 2026-06-23

**Authors:** Saeed Babaei, Matej Kanduč, Benoit Coasne, Mehdi Ostadhassan

**Affiliations:** † Civil Engineering Faculty, K. N. Toosi University of Technology, Tehran 1996715433, Iran; ‡ Department of Theoretical Physics, 61790 Jožef Stefan Institute, Jamova 39, Ljubljana 1000, Slovenia; § 27051 University Grenoble Alpes, CNRS, LIPhy, Grenoble 38000, France; ∥ Institut Laue Langevin, Grenoble F-38042, France; ⊥ Institute of Geosciences, Marine and Land Geomechanics and Geotectonics, 9179 Christian-Albrechts Universität, Kiel 24118, Germany; ¶ State Key Laboratory of Continental Shale Oil, Northeast Petroleum University, Daqing, 163318, China

## Abstract

Accurately predicting gas storage in shale requires understanding how adsorption alters the porous structure of kerogen, the main organic component of the rock. Gas adsorption can induce structural deformation in the microporous framework of kerogen, thereby modifying the observed excess adsorption isotherms. However, no comprehensive investigation has addressed how accessible volume evolves during adsorption. To address this gap, we employ a hybrid grand canonical Monte Carlo/molecular dynamics simulation of both immature and overmature amorphous kerogen matrices at 363.15 K and pressures up to 50 MPa, providing molecular-level insights into this adsorption-deformation process. Among the investigated gases, C_2_H_6_ displays the most pronounced reduction in excess adsorption at low pressures, attributed to its large molecular size. At higher pressures, excess adsorption decreases in the order CO_2_ > C_2_H_6_ > CH_4_ > N_2_, corresponding to roughly 50, 40, 30, and 20% reductions, respectively. Moreover, kerogen can undergo 2–9% strain at pressures up to 50 MPa, depending on gas type and kerogen structure. The Tóth model, which is a physical model that allows extending the Langmuir adsorption model to heterogeneous systems, further demonstrates that neglecting deformation-induced changes in accessible volume can lead to substantial errors when converting excess adsorption to absolute adsorption. These findings underscore the dynamic and deformable nature of kerogen, challenging the longstanding assumption of a rigid kerogen molecular structure. This has direct implications for accurately estimating gas-in-place in shale gas reservoirs and assessing storage capacity for subsurface carbon sequestration.

## Graphical abstract

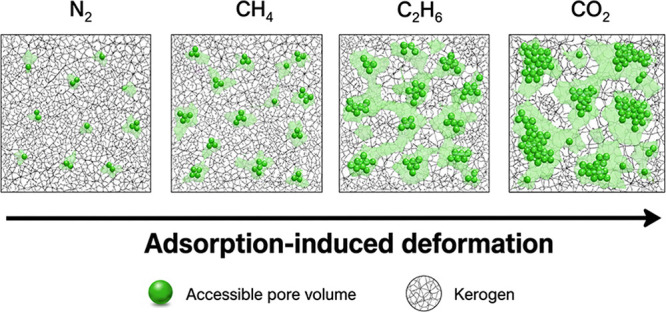

## Introduction

In contrast to conventional reservoirs, where hydrocarbons are primarily present as bulk-phase fluids (liquid or gas), shale reservoirs store most of their gas within nanoscale pores of organic matter (OM), mainly kerogen.^1,2^ Kerogen, the principal OM component, is characterized by its insolubility in common polar solvents and is a complex mixture consisting primarily of carbon (C), hydrogen (H), and oxygen (O), with smaller amounts of heteroatoms such as nitrogen (N) and sulfur (S).^3^ A notable feature of kerogen is its network of micropores,^4^ which can adsorb up to 85% of the gas present in shale formations.^5^

Gas adsorption has a significant impact on porous materials, especially when the pore size is comparable to the size of the gas molecule. Materials with pores smaller than 2 nm, known as microporous materials, are particularly affected.^6^ When molecules adsorb in these tiny pores, they interact with the pore walls, causing the material to deform, a phenomenon widely recognized as adsorption-induced deformation.^7–9^ Various factors, such as pore geometry, surface chemistry, and the physicochemical properties of the adsorbed fluid, influence the degree of deformation. Because of much smaller pore sizes, microporous materials experience stronger intermolecular forces than larger pore materials (mesoporous materials), leading to more substantial adsorption-induced stresses.

In the case of kerogen, the adsorption process can induce significant swelling, which leads to increased porosity and an expansion of the accessible volume.^10,11^ Thus, assuming a constant accessible volume when calculating excess adsorption is inappropriate where the pore structure evolves with adsorption.^12,13^ Instead, the adsorbed amount must be calibrated based on the accessible volume changes in the kerogen matrix. However, measuring adsorption-induced volume experimentally is challenging and time-consuming due to slight deformations and the complexity of extracting kerogen from shale samples.^13^ Therefore, using theoretical models to describe kerogen swelling becomes essential for a more accurate understanding of adsorption behaviors in shale reservoirs.

In recent years, molecular simulations have become a key tool to explore kerogen swelling in both gases and liquids. The primary techniques used include grand canonical Monte Carlo (GCMC) simulations, molecular dynamics (MD) simulations, and hybrid GCMC/MD simulations. In GCMC simulations, adsorbates (e.g., gas molecules) are stochastically inserted or removed from a fixed kerogen matrix to mimic exchange with a virtual bulk reservoir at a specified temperature and chemical potential (fugacity). The resulting adsorption behavior can then be used to compute volumetric changes, often through an extended poromechanical model.^14–16^ MD simulations, on the other hand, allow treating kerogen as deformable and compute swelling by initially equilibrating kerogen and in the presence of a fixed number of gas molecules in a constant-pressure (*NPT*) ensemble.^17^ However, this method cannot capture exchange with an external gas reservoir, making it difficult to directly relate the adsorption results to bulk reservoir conditions (e.g., pressure). The hybrid GCMC/MD approach combines the advantages of both methods: it alternates GCMC (to simulate gas exchange) and MD (to capture gas diffusion and matrix swelling), eliminating the need for predefined parameters such as the amount of gas in the kerogen matrix.^18,19^

In a direct comparison of GCMC/MD-*NVT* (performed at a constant volume)^20–22^ and GCMC/MD-*NPT* (performed at a constant pressure)^19,23,24^ approaches, Potier et al.^25^ examined argon adsorption and diffusion in a mature kerogen model. They found that although the GCMC/MD-*NVT* approach accounts for internal framework flexibility, it still underestimates adsorption capacity and diffusion coefficients compared to GCMC/MD-*NPT*, which allows for volume changes of the kerogen matrix in response to pressure and thermal volume fluctuations. When kerogen swells, the free volume increases, which leads to higher gas adsorption and diffusivity due to more space for molecules. This behavior aligns with the free volume theory,^26,27^ showing how changes in accessible volume affect adsorption and diffusion.

The results further suggest that the degree of kerogen swelling is directly related to the volume of adsorbed gas, with larger adsorbates causing less pronounced swelling.^28^ Importantly, models that ignore volume changes during swelling tend to underestimate adsorption capacity.^24,27,29^ Despite considerable efforts, previous GCMC/MD-*NPT* studies have mainly focused on the influence of kerogen swelling on absolute adsorption capacity and transport properties, while the evolution of accessible volume during adsorption remains poorly understood. In most studies, the accessible volume is implicitly assumed to remain constant and is commonly estimated using helium expansion methods.^30^ However, recent findings suggest that pore accessibility is highly sensitive to the choice of probe size, and should be defined based on the actual molecular size of the adsorbate, particularly in ultramicroporous kerogen structures.^31^ Moreover, adsorption-induced deformation can dynamically alter the accessible pore network during adsorption, potentially affecting excess adsorption behavior and pore size distribution (PSD). Despite this, no comprehensive study has systematically investigated the coupled relationship between kerogen swelling, accessible volume evolution, excess adsorption, and PSD across kerogen maturity levels under reservoir conditions.

To address this gap, the present study employs fully flexible hybrid GCMC/MD-*NPT* simulations to investigate adsorption-induced evolution of accessible volume in immature and overmature kerogen during the adsorption of CH_4_, C_2_H_6_, CO_2_, and N_2_ at 363.15 K and pressures up to 50 MPa. Unlike previous studies that primarily focused on absolute adsorption capacity, this work explicitly quantifies how swelling-induced changes in adsorbate-accessible porosity influence excess adsorption and PSD evolution using adsorbate-size-dependent accessibility analysis. The results provide new insights into the coupling between framework deformation, pore accessibility, and adsorption behavior in kerogen nanoporous structures under realistic shale reservoir conditions.

## Methodology

### Construction of Kerogen Matrices

All molecular simulations were performed using the LAMMPS software (stable released version, Aug 29, 2024).^32^ The kerogen matrices were built using 13 kerogen units of type IIA and 24 units of type IID, as proposed by Ungerer et al.^33^ Type IIA kerogen (C_252_H_294_O_24_N_6_S_3_) represents immature organic matter typically found in conventional oil and gas reservoirs, such as those in the North Sea, while type IID kerogen (C_175_H_102_O_9_N_4_S_2_) corresponds to overmature organic matter associated with unconventional gas formations like the Barnett Shale.

To model kerogen, we used the consistent valence force field (CVFF).^34^ The kerogen matrices were initially constructed by arranging the kerogen units within a cubic simulation cell (10 × 10 × 10 nm^3^) without overlap. The construction process followed the seven-stage procedure outlined in Table S2 of the Supporting Information (SI). To investigate the effect of PSD on adsorption, additional kerogen matrix models were generated by incorporating dummy particles (DPs) with a diameter of 2 nm into the matrices, creating larger pores. In these models, the DPs were removed after completing stage 7, and the systems were further relaxed in additional *NPT* simulations at 363 K and 0.1 MPa for 5 ns. Temperature and pressure were controlled using the Nosé–Hoover thermostat^35,36^ and Nosé–Hoover barostat^37,38^ with relaxation times of 100 and 1000 timesteps, respectively. An integration time step of 1 fs was employed to construct the kerogen structure. The Lorentz–Berthelot mixing rules were applied to calculate the Lennard-Jones (LJ) parameters for unlike interactions. A cutoff radius of 1.4 nm was set for nonbonded interactions without tail corrections.^31,39^ Long-range electrostatic interactions were computed using the particle–particle particle–mesh (PPPM) method with an accuracy of 10^–4^. Periodic boundary conditions were implemented in all three spatial dimensions.

In this study, kerogen type IIA-1 and type IID-1 refer to matrices without DPs, while kerogen type IIA-2 and type IID-2 correspond to matrices with DP-modified pores. Figure 1 presents the PSD and accessible volume for each kerogen model. The densities of kerogen type IIA-1 (1.14 g/cm^3^) and type IIA-2 (1.04 g/cm^3^) fall within the experimental range of 1.00–1.15 g/cm^3^ for type IIA kerogen.^40^ Similarly, densities of kerogen type IID-1 (1.31 g/cm^3^) and type IID-2 (1.22 g/cm^3^) align with the reported experimental range of 1.18–1.35 g/cm^3^ for type IID kerogen.^41^

**Figure 1:**
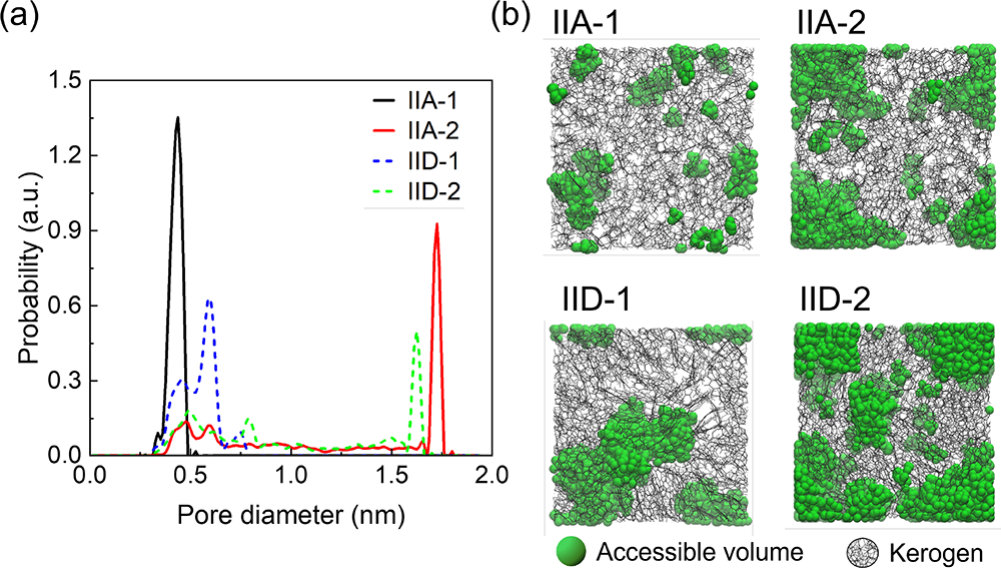
(a) Initial pore size distribution (at *P* = 0) in kerogen models. (b) Visualization of accessible volume in initial kerogen matrices using the CO_2_ probe. The visualization was generated with the VMD software.^42^

### Hybrid GCMC/MD Simulation

Recognizing that gas adsorption and kerogen deformability and swelling can profoundly influence each other, we investigated the adsorption of CH_4_, C_2_H_6_, CO_2_, and N_2_ gases using both rigid and flexible kerogen models under typical shale reservoir conditions (363.15 K and pressures up to 50 MPa). We used the TraPPE-small (all-atom) force field^43^ to simulate CO_2_ and N_2_ and the TraPPE-UA (united-atom) force field^44^ for CH_4_ and C_2_H_6_ molecules, which demonstrated both accuracy and computational efficiency in adsorption studies. The rigidity of C_2_H_6_, N_2_, and CO_2_ molecules was maintained using the Kamberaj et al.^45^ algorithm. All LJ parameters are summarized in Table S3. Previous studies have demonstrated good consistency between kerogen modeled with CVFF and gases such as CH_4_, C_2_H_6_, CO_2_, and N_2_ modeled with TraPPE force fields.^13,19–21,31,46^ To further support the reliability of this combination, we included a comparison of CH_4_ excess adsorption in flexible type IID kerogen with available experimental and simulation data in Figure S1.

The rigid kerogen was modeled as a static matrix by setting all atomic velocities to zero. These simulations were performed in the GCMC/MD-*NVT* ensemble, where gas molecules were inserted and deleted during the GCMC cycle, and their positions and orientations evolved during the MD step under constant volume conditions (*NVT* ensemble) using a time step of 1 fs. In contrast, flexible kerogen was simulated using the osmotic (GCMC/MD-*NPT*) ensemble. In this case, the same reservoir pressure was applied as the mechanical pressure acting on the kerogen matrix during the MD steps, which corresponds to drained conditions (which enables the observation of maximum deformation).^3^ Therefore, every 1000 fs of MD in the *NPT* ensemble, we conducted a GCMC cycle of 500 insertion and deletion attempts to simulate gas exchange. For the flexible model, a smaller time step of 0.5 fs was used to maintain numerical stability.

In GCMC simulations, the chemical potential μ is required as input. Similar to our previous study,^47^ we determined the dependence of gas pressure on chemical potential by performing additional simulations under bulk conditions (see Table S4). The GCMC simulation steps used the Metropolis algorithm,^48^ while the MD part applied the velocity Verlet algorithm^49^ to solve Newton’s equations. The total simulation time was 20 ns, with the final 5 ns dedicated to data analysis. Simulation input files are available in SI.

### Excess Adsorption

In this study, we follow the volumetric method,^50^ used in experimental studies, to calculate the excess adsorption isotherm:

$$n_{\mathrm{ex}}=n_{t}-\rho_{g}V_{\mathrm{acc}}$$ 1

where ρ_g_ is the bulk gas density, *V*_acc_ is the accessible volume, and *n*_ex_ and *n*_t_ are the excess and total adsorption amounts, respectively.

*V*_acc_ was determined using the probe-based method implemented in the PoreBlazer v4.0 software.^51^ In this method, a particle of a defined diameter interacts with the kerogen surface, generating a Connolly surface from which the accessible volume is calculated. The probe diameters were set at 0.373 nm for CH_4_, 0.410 nm for C_2_H_6_, 0.372 nm for N_2_, and 0.330 nm for CO_2_.^31^

## Results and Discussion

### Excess Adsorption

#### Rigid Kerogen

We begin with the analysis of excess adsorption in rigid kerogen models. As shown in Figure 2, the excess adsorption of gases in rigid type IIA and type IID kerogen strongly depends on thermal maturity. According to the van Krevelen diagram, the H/C and O/C ratios decreased with increasing kerogen maturity.^3^ Type IID kerogen shows significantly higher adsorption capacity than type IIA kerogen, primarily because of its greater aromatic content and porosity, which increase with thermal maturity.^52^ Larger porosity (Figure 1) leads to more accessible adsorption sites, resulting in higher adsorption capacity.

**Figure 2:**
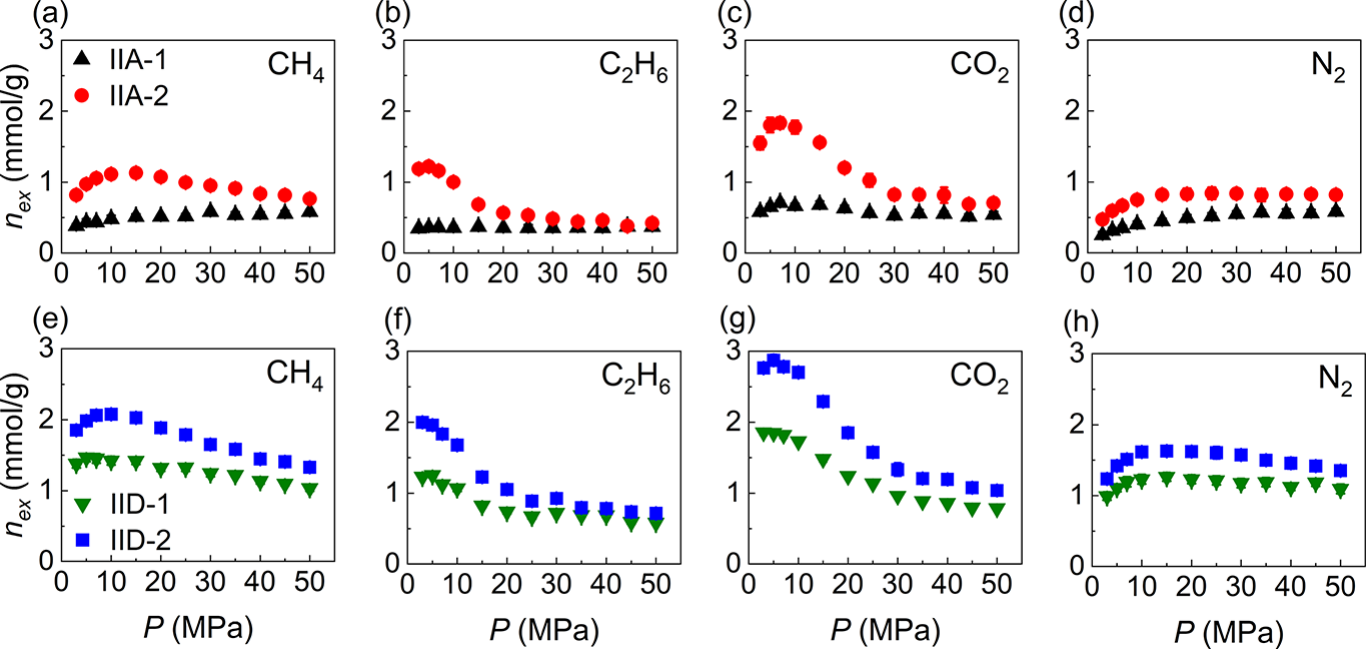
Excess adsorption isotherms of (a, e) CH_4_, (b, f) C_2_H_6_, (c, g) CO_2_, and (d, h) N_2_ in rigid type IIA and type IID kerogen matrices. Error bars are smaller than the symbols.

Excess adsorption exhibits a nonmonotonic dependence on pressure: it initially increases, reaches a maximum, and declines at very high pressures. This decline is a direct consequence of eq 1, since at pressures close to the critical region, the bulk gas density increases more rapidly with pressure than the adsorbed density. Notably, this behavior strongly depends on the ratio between the system temperature and the critical temperature *T*/*T*_c_. At temperatures far below *T*_c_, the vapor density is negligible and excess adsorption is expected to increase monotonically with pressure; in contrast, the nonmonotonic behavior observed here arises because the system temperature is close to or above *T*_c_.

To further investigate whether the higher adsorption value is related to molar volume or molecular interaction effects, we invoke the Gurvich rule^53^ which states that *n* ^sat^ = ρ_L_ *V*_acc_ where *n* ^sat^ is the adsorption uptake at saturation and ρ_L_ is the liquid molar density. ρ_*L*_ for CH_4_, C_2_H_6_, CO_2_, and N_2_ equal to 26.31, 18.09, 26.77, and 28.70 mmol/cm^3^, respectively.^31^ The ratios of maximum adsorption obtained from simulations to those predicted by the Gurvich rule for different fluids in rigid type IIA and type IID kerogen matrices are summarized in Table 1 (see Table S5 for corresponding values). For all kerogen matrices except type IIA-1 kerogen, the maximum adsorption values from simulation closely align with the predictions of the Gurvich rule, suggesting that adsorption is primarily governed by molar volume rather than molecular interactions. However, for type IIA-1 kerogen matrices, the maximum adsorption observed in this study significantly exceeds the value predicted by the Gurvich rule. As shown in Figure 1, most pores in the IIA-1 kerogen are small and isolated. In such highly confined pores, molecular geometry may influence pore accessibility. Linear molecules such as CO_2_ and N_2_, as well as dumbbell-shaped molecules such as C_2_H_6_, may access narrow pores differently depending on their orientation within the pore space. Because the accessible volume was evaluated using a spherical probe, these shape-dependent accessibility effects were not explicitly considered, which may contribute to deviations between the simulated adsorption capacities and the predictions of the Gurvich rule (see SI for further details). Therefore, for amorphous materials with extremely small pores, such as type IIA-1 kerogen, advanced pore structure characterization methods that explicitly account for molecular shape and orientation effects^54^ may be required to evaluate pore accessibility more accurately.

**Table 1:** Ratio of Maximum Adsorption (Simulation/Gurvich Rule) for Different Fluids in Rigid Type IIA and Type IID Kerogen Matrices

fluid	IIA-1	IIA-2	IID-1	IID-2
CH_4_	2.49	1.27	1.12	0.90
C_2_H_6_	4.51	1.75	1.33	1.09
CO_2_	1.55	1.67	1.09	0.97
N_2_	2.06	0.87	1.02	0.77

In Figure S2, the maximum adsorption values from the simulations are plotted against the liquid molar densities of the corresponding gases. Although a linear relationship between adsorption capacity and liquid molar density (*n* ^sat^ ∝ ρ_L_) may be expected based on the Gurvich rule, such proportionality strictly holds only if the accessible volume remains constant for all adsorbates. The observed deviations from linearity therefore indicate that both molecular accessibility and fluid–solid interactions play a significant role.

In particular, N_2_ exhibits a lower saturation capacity than expected from its liquid molar density. This behavior cannot be attributed solely to its linear geometry, since CO_2_ is also linear. Instead, the reduced adsorption of N_2_ is primarily due to its weaker interactions with the kerogen matrix, combined with steric limitations that restrict efficient packing within the pore space.

Among the investigated gases, CO_2_ shows the highest adsorption capacity, exceeding CH_4_, C_2_H_6_, and N_2_. This enhanced adsorption arises from the combined effects of strong fluid–solid interactions, associated with its significant quadrupole moment, and its ability to achieve relatively efficient packing within confined pores. The pressure-dependent behavior further highlights the competition between interaction strength and packing efficiency. At low pressures, C_2_H_6_ adsorbs more strongly than CH_4_ due to stronger dispersive interactions. However, at higher pressures, CH_4_ exceeds C_2_H_6_ because its smaller molecular size allows more efficient packing within the available pore volume. Overall, these observations indicate that the maximum adsorption capacity is governed not only by liquid molar density but also by molecular size, shape, and interaction strength.

In contrast, fluid–solid and intermolecular interactions primarily determine the adsorption affinity and the uptake behavior at subsaturation pressures. For example, although CH_4_ and N_2_ have comparable molecular sizes, CH_4_ exhibits significantly higher adsorption across the entire pressure range, consistent with its much larger LJ well depth (ε/*k*_B_ = 148 K) compared to N_2_ (ε/*k*_B_ = 36 K).

#### Flexible Kerogen

Having established the behavior in rigid kerogen, we next examine the effects of matrix flexibility, where adsorption-induced deformation is explicitly taken into account. In such systems, deformation alters the internal structure and accessible space within the matrix, which in turn affects how excess adsorption should be calculated. To evaluate excess adsorption in this context (eq 1), we used two approaches: one based on a fixed accessible volume, *V*_acc_^f^, and the other on a variable accessible volume, *V*_acc_^v^. In the fixed-volume approach, *V*_acc_^f^ is computed only from the initial unloaded structure at zero pressure and is then used at all conditions. In contrast, in the variable-volume approach, *V*_acc_^v^ is dynamically updated for each configuration in response to adsorption-induced deformation.

Figure 3 shows that excess adsorption computed from the flexible models is smaller when using the variable-volume approach because accessible volume increases with adsorption. For instance, in type IIA kerogen, the relative mismatch between the two approaches at 10 MPa is 6%, 11%, 11%, and 5% for CH_4_, C_2_H_6_, CO_2_, and N_2_, respectively. At 50 MPa, these differences increase markedly to 53%, 20%, 62%, and 26%. Similarly, for type IID kerogen, the mismatches at 10 MPa are 5%, 13%, 6%, and 3%, respectively, and rise dramatically to 91%, 58%, 78%, and 20% at 50 MPa. The discrepancies become more pronounced at higher pressures, supporting the notion that neglecting adsorption-induced deformation and changes in accessible volume can lead to substantial errors in estimations of excess adsorption.

**Figure 3:**
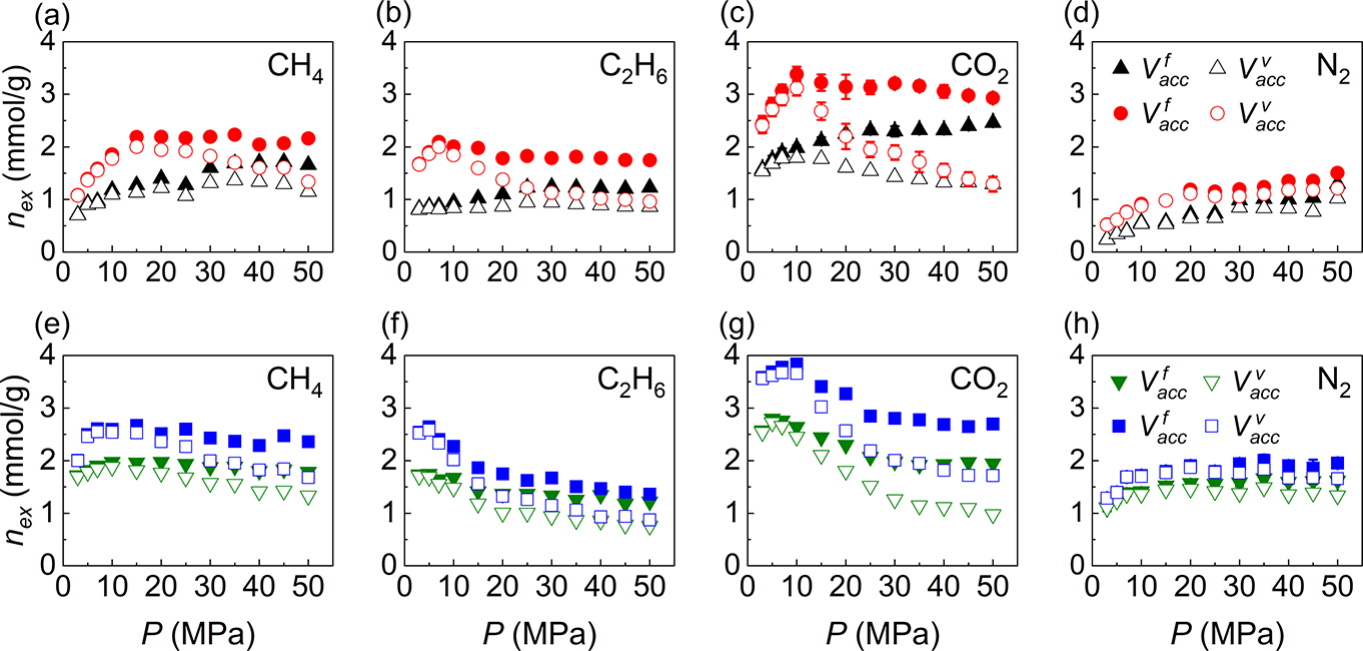
Excess adsorption isotherms of CH_4_, C_2_H_6_, CO_2_, and N_2_, calculated using both fixed (*V*_acc_^f^) and variable (*V*_acc_^v^) accessible volume definitions in flexible type IIA (a–d) and type IID (e–h) kerogen matrices. Symbols denote different kerogen models: IIA-1 (triangle), IIA-2 (circle), IID-1 (inverted triangle), and IID-2 (square).

Excess adsorption depends on the balance between accessible volume and the number of adsorbed molecules (eq 1). Specifically, increasing the *V*_acc_ lowers excess adsorption, while a higher number of adsorbed molecules, *n*_t_, increases overall adsorption within the pores. Recent studies^39,55^ demonstrated that even minor variations in the accessible volume can significantly influence the trend of excess adsorption with pressure, as shown in Figure 3.

To better understand the relative differences between the results from the fixed and variable volume approaches, Figure 4 shows the ratio of excess adsorption computed using *V*_acc_^v^ to that computed using *V*_acc_^f^. As pressure increases, the difference between the two values grows, with particularly steep changes observed beyond 20 MPa. This finding indicates that, while most laboratory experiments are limited to pressures around 15 MPa due to equipment constraints, extrapolating excess adsorption data beyond this range using conventional adsorption models may lead to inaccuracies. All our kerogen models exhibit similar trends across different gases, with the exception of CH_4_ in IID-2, which shows deviations at high pressures. At low pressures, the most significant reductions in excess adsorption is exhibited by CO_2_ and C_2_H_6_. This volume reduction for C_2_H_6_ is likely due to its larger molecular size, though the effect stabilizes at higher pressures because the pores become saturated. In contrast, the other gases show a progressively declining trend in adsorption ratios at all pressures, with CO_2_ experiencing the greatest reduction at very high pressures, followed by CH_4_ and N_2_. Even for N_2_, which has a relatively low adsorption capacity, the discrepancy between the two volume assumptions can be as high as 20% at 50 MPa. This highlights the potential for significant errors if these variations are not taken into account.

**Figure 4:**
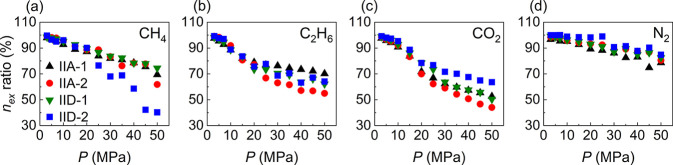
Excess adsorption ratio as a function of pressure for (a) CH_4_, (b) C_2_H_6_, (c) CO_2_, and (d) N_2_ in flexible type IIA and type IID kerogen matrices. The excess adsorption ratio is defined as the excess adsorption calculated using *V*_acc_^v^ relative to that calculated using *V*_acc_^f^ in flexible kerogen.

### Accessible Volume

To further elucidate how accessible volume influences excess adsorption, we present the variation of accessible volumes with pressure (Figures S3 and S4) and their corresponding ratios (Figure 5). Among all models, IIA-1 experiences the largest relative changes in pore volume (Figure 5), especially with C_2_H_6_, which induces the most significant swelling owing to its larger molecular size compared to the other gases. However, as seen in Figure S3, the pore volume stabilizes at higher pressures due to constraints imposed by pore size and the molecular dimensions of C_2_H_6_. With the exception of IIA-1, the overall trend in accessible volume change follows the order CO_2_ > C_2_H_6_ > CH_4_ > N_2_. Notably, despite the comparable molecular sizes of CH_4_ and N_2_, CH_4_ has a higher affinity to kerogen, leading to higher adsorption and a greater volume change. Meanwhile, even though CO_2_ is the smallest molecule, it causes significant volume changes, owing to its strong interaction with kerogen, which leads to the highest adsorption.

**Figure 5:**
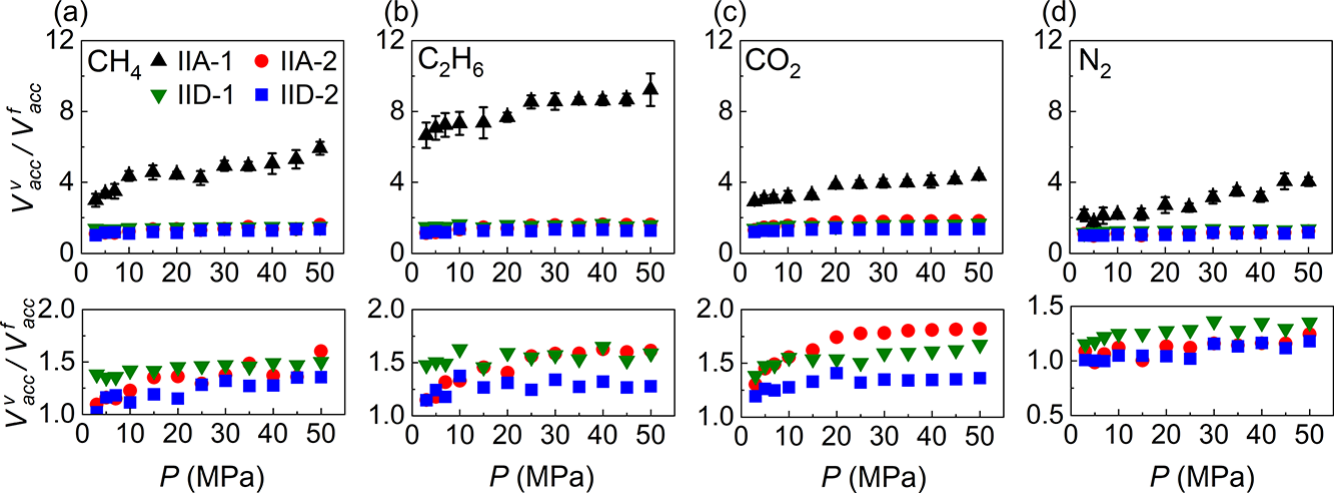
Accessible volume ratio, *V*_acc_^v^/*V*_acc_^f^ as a function of pressure for (a) CH_4_, (b) C_2_H_6_, (c) CO_2_, and (d) N_2_ in flexible type IIA and type IID kerogen matrices. Lower panels correspond to zoomed-in representations of the upper panels for improved visualization.

The relationship between the amount adsorbed and the accessible volume at constant pressure was further investigated, as shown in Figure S5. An approximately linear correlation is observed between adsorption and accessible volume, indicating that, under the specific conditions and models studied here, pore space availability plays a key role in governing adsorption, with increasing pore volume generally corresponding to higher adsorption capacity. This trend is consistently observed across different gases within the investigated pressure range, reflecting a robust coupling between adsorption and accessible volume in the flexible kerogen matrices. However, differences in adsorption capacities among gases highlight the influence of distinct molecular interactions and affinities with the kerogen matrix.

### Absolute Adsorption

Although experiments commonly measure excess adsorption, absolute adsorption provides a more accurate measure of the total amount of gas retained in the material. Unlike experiments, molecular simulations allow for direct calculation of absolute adsorption without relying on assumptions about the adsorbed phase density.

Figure 6 presents the absolute adsorption values obtained from our simulations. The maximum adsorption (i.e., the adsorption capacity) across both rigid and flexible kerogen matrices follows the order IIA-1 < IID-1 < IIA-2 < IID-2 (Figure 6a–d), which largely reflects the trend of increasing PSD (Figure 1a). The only exception is a small reversal between IIA-2 and IID-2. As expected, matrix flexibility increases adsorption capacity, following the reverse trend: IID-2 < IID-1 < IIA-2 < IIA-1 (Figure 6e–h). This implies that the largest error from neglecting flexibility occurs in low-porosity matrices with low adsorption capacity. For example, in IIA-1, neglecting flexibility leads to a 3-fold underestimation. Conversely, for high-porosity, high-capacity matrices, flexibility still matters, but to a lesser extent; for instance, in IID-2, the discrepancy is around 40%.

**Figure 6:**
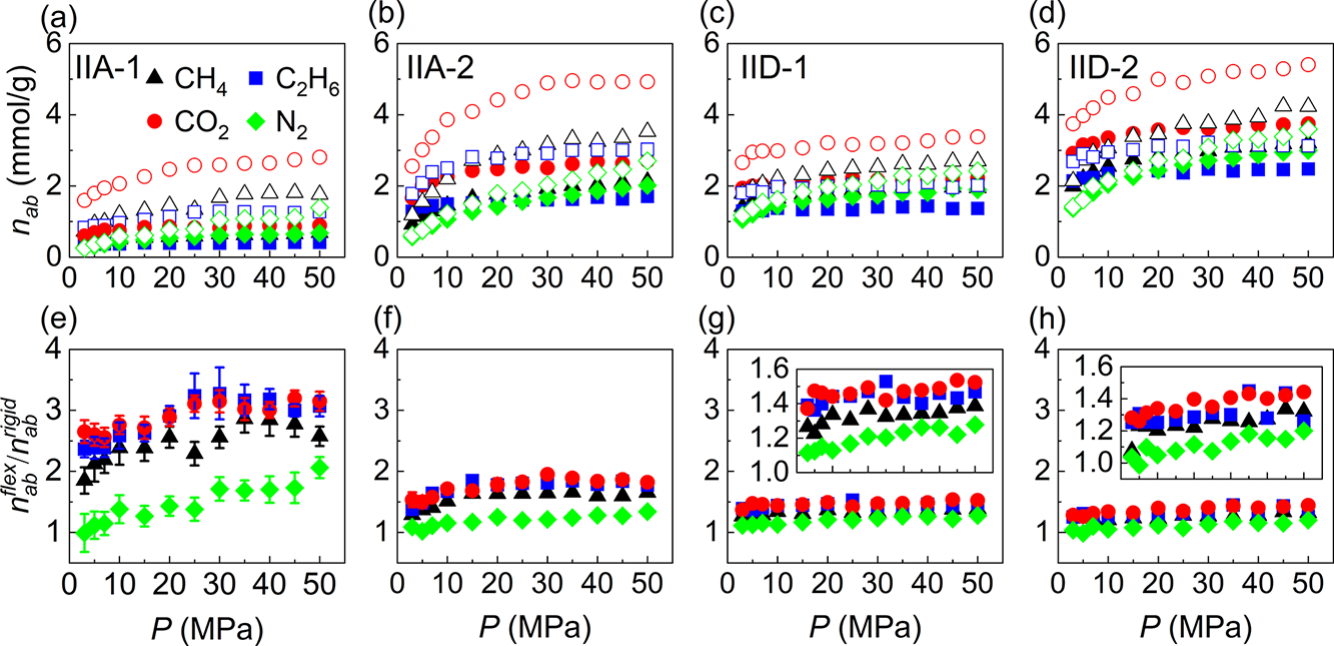
Absolute adsorption of gases as a function of pressure in rigid (solid) and flexible (open) kerogen matrices for (a) IIA-1, (b) IIA-2, (c) IID-1, and (d) IID-2. Adsorption ratios as functions of pressure, defined as the amount of gas adsorbed in flexible kerogen relative to rigid kerogen, for (e) IIA-1, (f) IIA-2, (g) IID-1, and (h) IID-2.

Finally, when comparing adsorption capacities for different gases across all matrices, we observe the following descending trend: CO_2_ > CH_4_ > C_2_H_6_ > N_2_. This order does not correlate directly with molecular size or adsorption affinity, suggesting a more complex interplay of molecular interactions and pore confinement effects.^13,28^ Our results indicate that matrix flexibility leads to higher excess and absolute adsorptions, with the extent of swelling influenced by kerogen maturity and pore structure. We now turn to analyze how this swelling impacts the volume of the entire kerogen matrix.

### Volume Response

Gas adsorption also causes the entire matrix to expand, altering its porosity and storage capacity. To quantify this effect, we define the volumetric strain, ε_*v*_, as a relative volume expansion:

$$\varepsilon_{v}=\frac{V-V_{0}}{V_{0}}$$ 2

where *V* and *V*_0_ are the average matrix volumes after and before adsorption, respectively. In the literature, ε_v_ is typically modeled as a linear function of the adsorption amount.^19,24,25,27^ Nonetheless, changes in pore volume caused by adsorption-induced strain may inversely influence adsorption quantities, resulting in a complex interdependency. Studies also observed a nonlinear relationship between ε_*v*_ and gas adsorption, employing a quadratic function to capture nonlinear effects.^56,57^

Figure 7 represents the relationship between volumetric strain and gas adsorption as obtained from our simulations. The results demonstrate that the relationship can be well described by either linear or quadratic functions, consistent with previous research on kerogen.^19,24,57^ Notably, at 50 MPa, CH_4_, C_2_H_6_, CO_2_, and N_2_ induce maximum ε_v_ of 5.6%, 6.3%, 8.7%, and 2.8%, respectively, depending on kerogen maturity and PSD. The quadratic fits for gases in kerogen (Figure 7) show slight shrinkage of the kerogen at low adsorption values (i.e., low pressures), consistent with previous reports on H_2_ and CO_2_ adsorption in kerogen.^19,27^ This behavior arises because, at low pressures, the kerogen cavities deform to enhance interactions with the gas molecules, then it increases as adding adsorbed molecules requires making room in the system.^58^ Therefore, linear or quadratic functions appear suitable for approximating the relationship between adsorption-induced strain and adsorption amounts.

**Figure 7:**
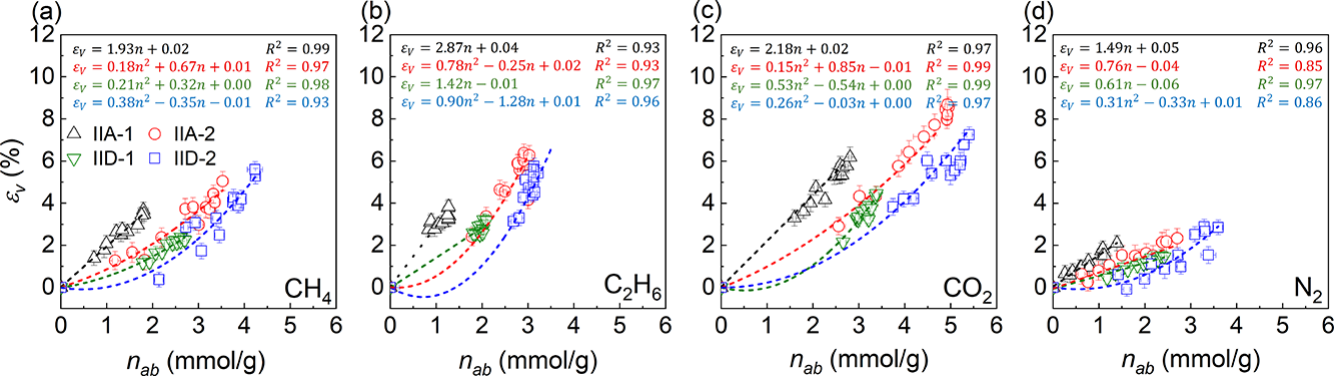
Volumetric strain as a function of adsorption for (a) CH_4_, (b) C_2_H_6_, (c) CO_2_, and (d) N_2_ in type IIA and type IID kerogen matrices.

Furthermore, the effect of PSD is reflected in the amount of gas accommodated within the pore space. Systems with larger pores and broader PSD exhibit higher adsorption at a given pressure, resulting in greater volumetric strain at comparable conditions. This trend is evident in Figure 7, where kerogen models with larger pores show higher adsorption levels at similar strain values. Interestingly, when comparing data points for different gases in the same kerogen sample, we observe that volumetric strain depends primarily on gas adsorption, with only a minor influence of gas type. These findings have important implications for gas storage capacity, transport behavior, and recovery efficiency in organic-rich shale formations.

To further investigate whether the observed increase in accessible volume can be partially or fully attributed to matrix volume expansion, their relationship is analyzed and plotted in Figure 8. The results reveal an approximately linear correlation between these two volumes, closely following a slope of 1. This suggests that volume expansion significantly contributes to, and possibly entirely accounts for, the observed increase in accessible volume.

**Figure 8:**
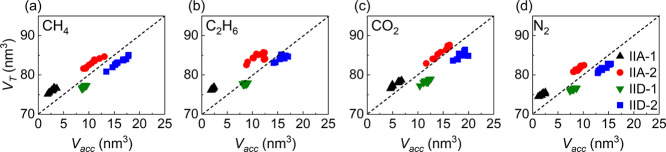
Total volume (i.e., simulation box volume) as a function of accessible volume for (a) CH_4_, (b) C_2_H_6_, (c) CO_2_, and (d) N_2_ in type IIA and type IID kerogen matrices across the full range of applied pressures. The dashed line indicates a slope of 1.

### Excess to Absolute Adsorption Conversion Methods

Our molecular simulation data allow us to evaluate adsorption models. Given that experimental research can only directly quantify excess adsorption, we convert excess adsorption to absolute adsorption by applying the standard correction based on adsorbed phase density, defined by

$$n_{\mathrm{ex}}=n_{\mathrm{ab}}\left(1-\frac{\rho_{g}}{\rho_{\mathrm{ad}}}\right)$$ 3

where *n*_ab_ is the amount of absolute adsorption, ρ_g_ is the bulk gas density, and ρ_ad_ is the density of the adsorbed phase. While ρ_g_ is known from thermodynamic data, ρ_ad_ cannot be directly measured, making its estimation a key challenge in adsorption modeling.^39^ Nevertheless, the freely fitting method is frequently employed, which involves fitting a theoretical adsorption isotherm model to experimental excess adsorption data, treating ρ_ad_ as a fitting parameter. Under this approach, a modified model is calibrated against the excess adsorption isotherm to determine the adsorption phase density. Common models, such as Langmuir, Tóth, and supercritical Dubinin–Radushkevich (SDR), are used to convert excess adsorption to absolute adsorption in shale gas.^47,50^ In this study, we used the Tóth model,^59^ which is a physical model that allows extending the Langmuir adsorption model to a heterogeneous system:

$$n_{\mathrm{ab}}^{\mathrm{Toth}}=\frac{n_{\max}kP}{[1+(kP{)}^{t}{]}^{1/t}}$$ 4

where *n*_ab_^Toth^ represents the absolute adsorption (i.e., loading) at various equilibrium pressures, *n*_max_ is the theoretical maximum adsorption capacity, *P* is pressure, *t* reflects the heterogeneity of the adsorbent, and *k* is the Tóth equilibrium constant, reflecting the affinity between the adsorbate and adsorbent, higher *k* values indicate stronger binding.

The fitted parameters are provided in Table S6. In this study, the adsorbed density sometimes exceeds the bulk liquid density. This apparent behavior arises from the strongly nonlinear nature of adsorption isotherms with respect to their fitting parameters, which permits multiple parameter combinations to adequately reproduce excess adsorption data. Nevertheless, it is important to emphasize that not all fitted parameter sets are physically meaningful or consistent with realistic thermodynamic constraints.^60^

Figure 9 compares the absolute adsorption values predicted by the Tóth model with those obtained from simulations. The predictions by the Tóth model become worse for larger pore sizes and broader pore distributions (solid lines). However, incorporating variable volume adjustments (dashed lines) generally leads to better agreement with simulation results. This improvement highlights the critical importance of accounting for changes in accessible volume when converting measured excess adsorption to absolute adsorption for accurate characterization.

**Figure 9:**
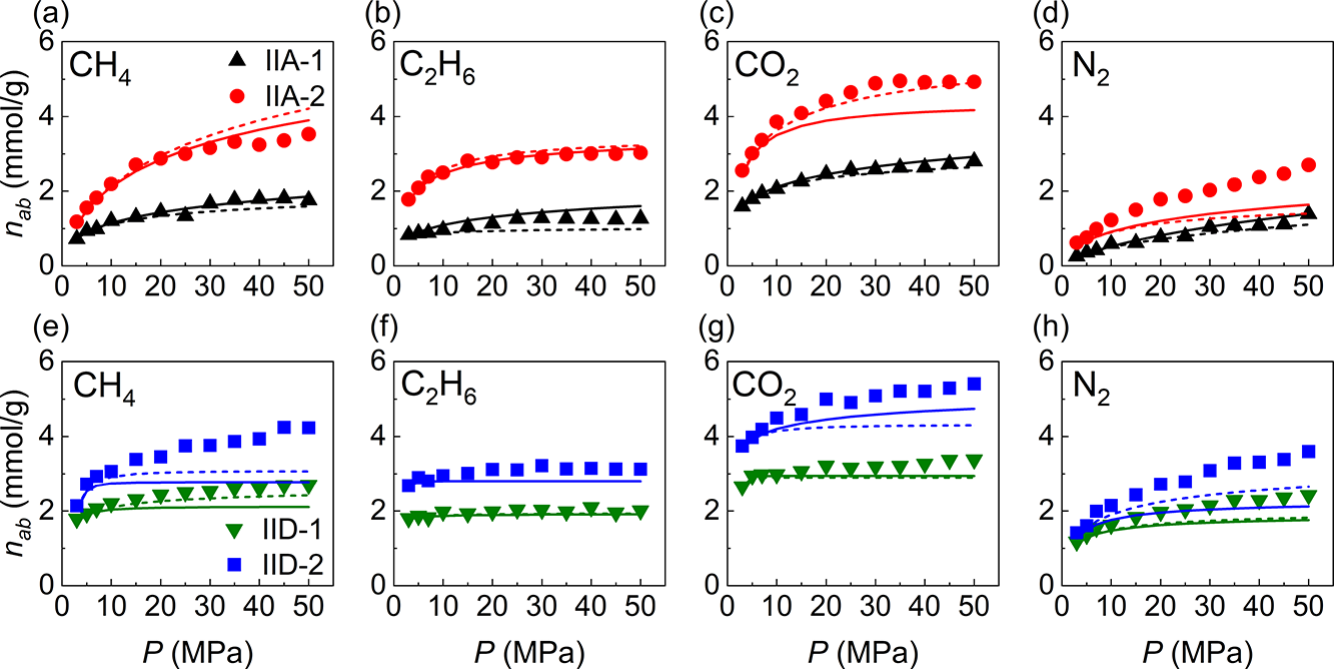
Comparison of absolute adsorption predictions from the Tóth model and molecular simulations for flexible type IIA (a–d) and type IID (e–h) kerogen matrices for CH_4_, C_2_H_6_, CO_2_, and N_2_. Results are shown using two accessible volume definitions: variable accessible volume, *V*_acc_^v^ (solid line), and fixed accessible volume, *V*_acc_^f^ (dashed line). Error bars are smaller than the symbols.

## Conclusions

Using a hybrid GCMC/MD-*NPT* approach, we investigated adsorption-induced deformation in immature and overmature kerogen samples at *T* = 363.15 K and pressures up to 50 MPa, considering two distinct pore size distributions. The main conclusions are as follows:

1. Adsorption-induced swelling consistently reduces excess adsorption, with the deviation becoming more pronounced at higher pressures as pore expansion increases.
2. Neglecting kerogen flexibility leads to substantial overestimation of adsorption, reaching up to a factor of ∼3 in low-porosity systems and remaining on the order of ∼ 40% in higher-porosity structures.
3. The magnitude of swelling strongly depends on pore size, with the largest deformation occurring in smaller pores and progressively smaller changes in larger pores.
4. Immature kerogen exhibits greater swelling than overmature kerogen under comparable conditions.
5. Depending on the kerogen structure and gas type, the relationship between adsorption and deformation follows either a linear or quadratic trend.
6. Accounting for a variable accessible volume improves the performance of the Tóth model in converting excess to absolute adsorption, compared to the conventional fixed-volume assumption.

Overall, neglecting adsorption-induced pore deformation can introduce significant systematic errors in adsorption estimates. These findings highlight the importance of accounting for such deformation when interpreting adsorption behavior in organic-rich shales, with direct implications for experimental analysis, model development, and gas-in-place estimation. Future work should extend this framework to multicomponent systems and further examine the coupling between deformation, molecular transport, and confinement effects across different kerogen types and maturities.

## Supplementary Material





## Notes

Sensitivity of accessible volume; comparison of CH_4_ excess adsorption with other studies; maximum adsorption capacities of different gases; accessible and fixed volumes in kerogen at different pressures; pressure effect on accessible volume in flexible kerogen; accessible volume vs pressure and adsorption in kerogen; molecular dynamics procedure for kerogen matrix creation; Lennard–Jones parameters; chemical potentials used for GCMC simulations; comparison of adsorption from the Gurvich rule and simulations; and fitting parameters for the Tóth model (PDF)
LAMMPS input files (ZIP)
