# Supplementary material for: Adsorption-Induced Pore Volume Deformation: Implications for Excess Adsorption in Kerogen Matrices
Source: Langmuir. 2026 Jun 23;42(26):18757–66. doi: 10.1021/acs.langmuir.6c00872 (PMC13352615; doi:10.1021/acs.langmuir.6c00872)
Supplement: Supplementary file 1 [file la6c00872_si_001.pdf]

Supporting Information: Adsorption-Induced Pore Volume Deformation: Implications for Excess Adsorption in Kerogen Matrices

Saeed Babaei,[†] Matej Kanduč,[*,‡] Benoit Coasne,[¶,§] and Mehdi Ostadhassan[*,‖,⊥]

†*Civil Engineering Faculty, K. N. Toosi University of Technology, Tehran 1996715433, Iran*

‡*Department of Theoretical Physics, Jožef Stefan Institute, Jamova 39, Ljubljana, 1000, Slovenia*

¶*University Grenoble Alpes, CNRS, LIPhy, 38000 Grenoble, France*

§*Institut Laue Langevin, F-38042 Grenoble, France*

‖ *State Key Laboratory of Continental Shale Oil, Northeast Petroleum University, Daqing, 163318, China*

⊥*Institute of Geosciences, Marine and Land Geomechanics and Geotectonics, Christian-Albrechts Universität, Kiel 24118, Germany*

E-mail: matej.kanduc@ijs.si; mehdi.ostadhassan@nepu.edu.cn,mehdi.ostadhassan@ifg.uni-kiel.de

Table of Contents

Sensitivity of accessible volume S2

Comparison of CH_4 excess adsorption with other studies S3

Maximum adsorption capacities of different gases S4

Accessible and fixed volumes in kerogen at different pressures S5

Pressure effect on accessible volume in flexible kerogen S5
Accessible volume vs. pressure and adsorption in kerogen S6
Molecular dynamics procedure for kerogen matrix creation S6
Lennard–Jones paramters S6
Chemical potentials used for GCMC simulations S7
Comparison of adsorption from the Gurvich rule and simulations S7
Fitting parameters for the Tóth model S8
References S9

Sensitivity of accessible volume

To account for the non-spherical geometry of N_2, CO_2, and C_2H_6, a shape-sensitivity analysis was conducted to quantify the systematic uncertainty inherent in the conventional spherical probe approximation (Table S1). For each species, the accessible volume V_{acc} was bounded by two limiting scenarios defined by the minimum (a) and maximum (b) molecular dimensions. These parameters were derived from the TraPPE force field[1,2] as follows: C_2H_6 ($a = 3.75, b = 5.29$ Å), CO_2 ($a = 3.05, b = 5.37$ Å), and N_2 ($a = 3.31, b = 4.41$ Å). This dual-diameter approach establishes the physical range of accessibility by accounting for the orientation-dependent exclusion of linear molecules such as CO_2 and N_2, as well as dumbbell-shaped molecules such as C_2H_6, within the highly confined kerogen geometries.

As illustrated by Table S1, the accessibility in matrices with larger pore systems (e.g., IIA-2 and IID-2) remains relatively robust against shape variations, showing only minor variations. However, in the ultra-microporous IIA-1 matrix (LCD = 4.66 Å), a significant orientation-dependent exclusion is observed.

For C_2H_6 and CO_2, the V_{acc} values drop to zero when the probe diameter is set to the maximum dimension b. This physical trend indicates that these molecules can only traverse the constrained pore network when favorably aligned with their narrowest cross-

Table S1: Sensitivity of accessible volume V_{acc} (cm^3/g) to molecular dimensions a and b. The largest cavity diameters (LCD) for IIA-1, IIA-2, IID-1, and IID-2 matrices are 4.66, 17.49, 7.53, and 16.51 Å, respectively.

Structure	Fluid	V_{acc} (Standard)	V_{acc} (Probe a)	V_{acc} (Probe b)
IIA-1	C_2H_6	0.005	0.009	0.000
	CO_2	0.021	0.029	0.000
	N_2	0.011	0.081	0.002
IIA-2	C_2H_6	0.095	0.100	0.081
	CO_2	0.110	0.110	0.080
	N_2	0.101	0.109	0.091
IID-1	C_2H_6	0.057	0.065	0.037
	CO_2	0.076	0.085	0.036
	N_2	0.065	0.076	0.002
IID-2	C_2H_6	0.125	0.132	0.104
	CO_2	0.144	0.150	0.102
	N_2	0.134	0.143	0.119

section (dimension a). These calculated ranges, detailed in Table S1, provide a comprehensive envelope for the potential systematic errors introduced by the standard spherical probe method and highlight the critical role of molecular orientation in highly confined kerogen environments.

Validation

The CH_4 excess adsorption isotherm obtained from molecular simulation for type IID kerogen is compared with experimental data[3,4] and previous simulation studies,[5,6] as shown in Figure S1. Overall, the simulated results show reasonable agreement with both experimental and literature data. Small discrepancies between molecular simulations and experiments can be attributed to the complexity of natural samples used in experiments, which typically contain various mineral components and pores with heterogeneous shapes and size distributions. In contrast, molecular simulations are performed on simplified models due to computational limitations, where only selected pore geometries, kerogen structures, and compositions are considered. Moreover, differences among simulation studies can arise from variations in

temperature conditions and the number of kerogen units, which directly affect the resulting pore size distribution and adsorption behavior.

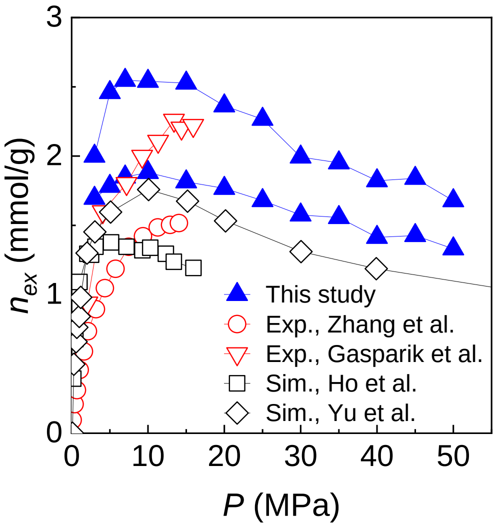

Figure S1: Comparison of the calculated CH_4 excess adsorption isotherms in flexible type IID kerogen at 363 K with experimental[3,4] and simulation[5,6] results.

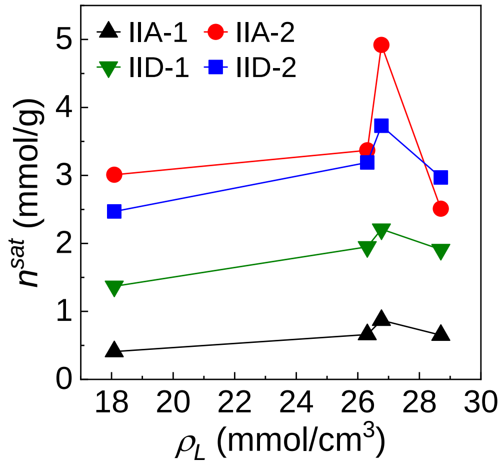

Figure S2: Comparison of maximum adsorption capacities for different gases from molecular simulations at P= 50 MPa as a function of liquid molar density ρ_L in rigid type IIA and type IID kerogen matrices. ρ_L for CH_4, C_2H_6, CO_2, and N_2 equal to 26.31, 18.09, 26.77, and 28.70 mmol/cm^3, respectively.[7]

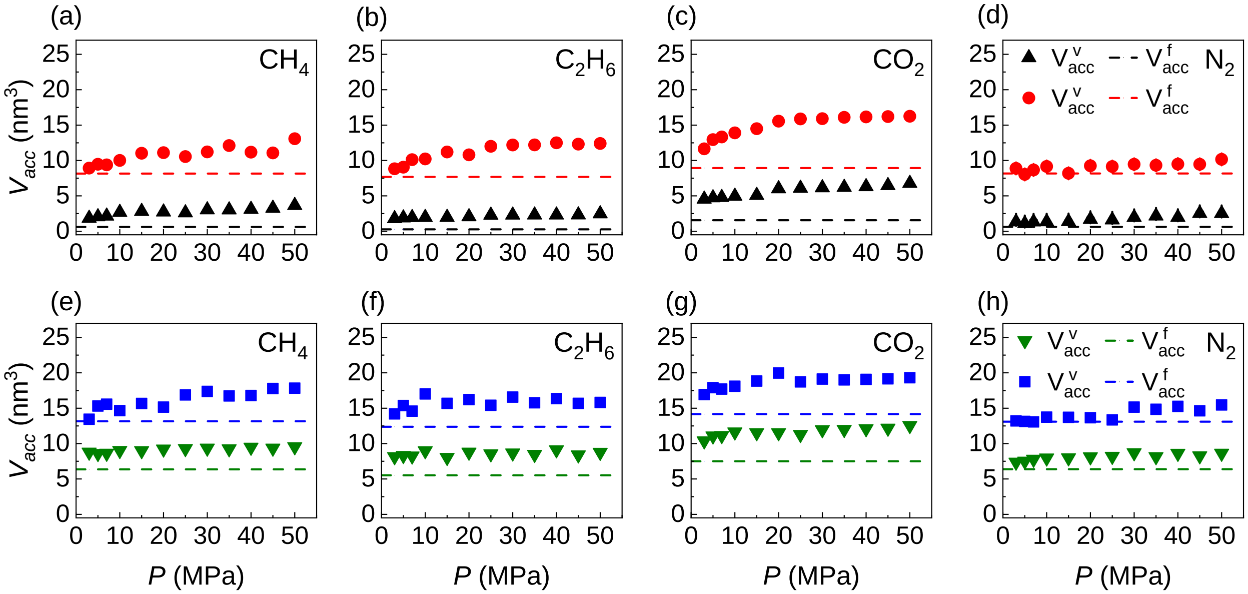

Figure S3: Variable (V^v_{acc}) and fixed (V^f_{acc}) accessible volumes as a function of pressure for CH_4, C_2H_6, CO_2, and N_2 in flexible type IIA (a–d) and type IID (e–h) kerogen matrices. Symbols denote different kerogen models: IIA-1 (triangle), IIA-2 (circle), IID-1 (inverted triangle), and IID-2 (square).

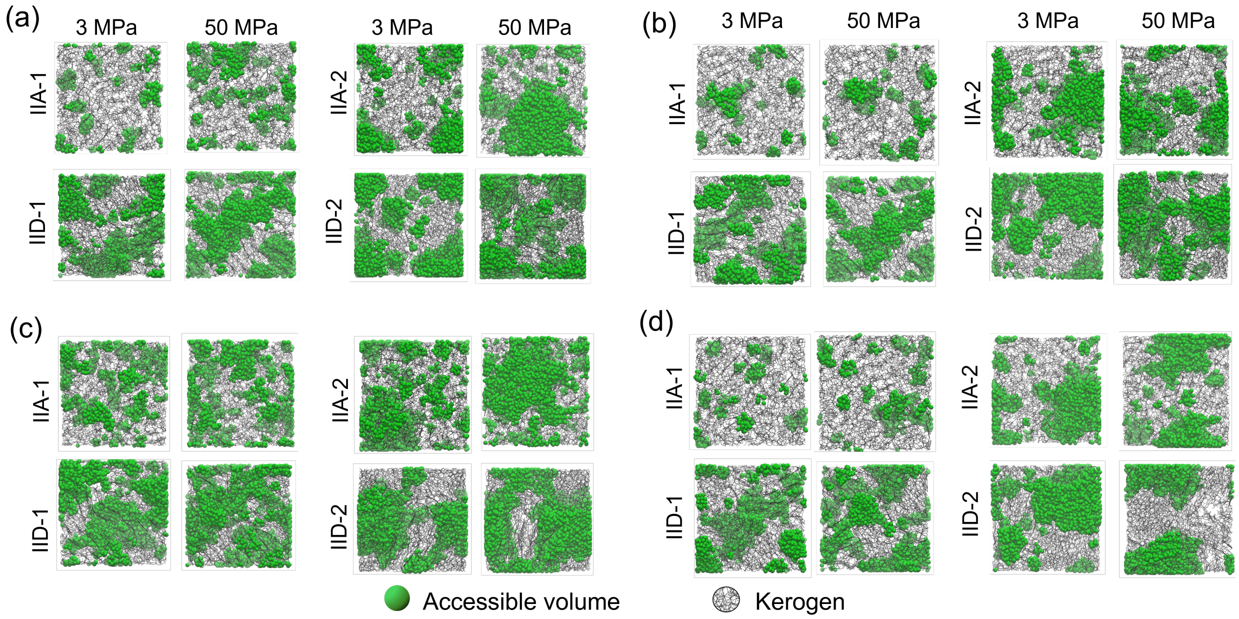

Figure S4: Visualization of accessible volumes as a function of pressure for flexible type IIA and type IID kerogen matrices (a) CH_4, (b) C_2H_6, (c) CO_2, and (d) N_2.

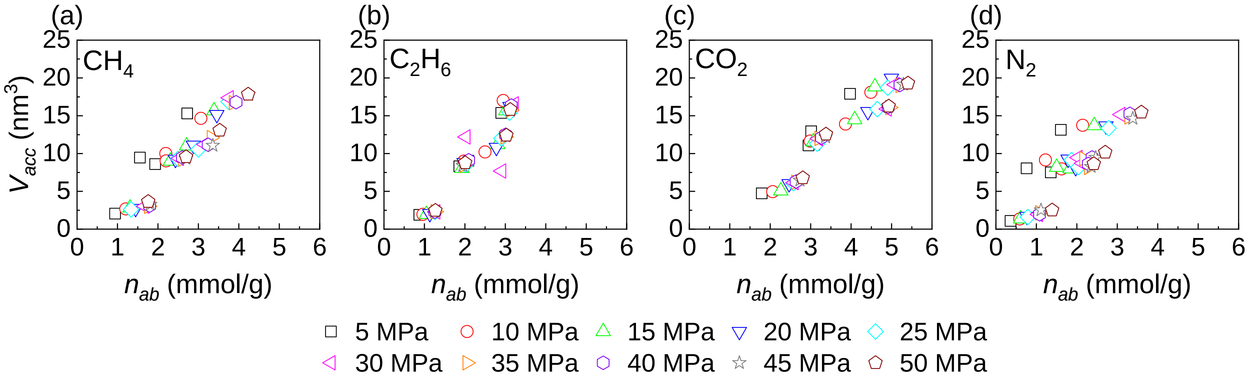

Figure S5: Accessible volume as a function of adsorption at different pressures for (a) CH_4, (b) C_2H_6, (c) CO_2, and (d) N_2 in flexible type IIA and type IID kerogen matrices.

Table S2: Molecular dynamics relaxation procedure to create the kerogen matrix. For Models 2 and 3, dummy particles (DPs) with diameters of 1 and 2 nm, respectively, were used in the simulations.

Stage	Ensemble	T (K)	P (MPa)	Time (ns)
1	NVT	900	-	0.3
2	NPT	900	20	0.3
3	NPT	900 $\rightarrow$ 700	20	0.3
4	NPT	700 $\rightarrow$ 500	20	0.3
5	NPT	500 $\rightarrow$ 300	20	0.3
6	NPT	300	20 $\rightarrow$ 0.1	0.3
7	NPT	300	0.1	10

Table S3: Lennard-Jones (LJ) and partial charge parameters for gas molecules.[1,2]

Molecule	(pseudo) atom	σ (nm)	ϵ/k_B (K)	q (e)
CH_4	CH_4	0.373	148.0	0.000
C_2H_6	CH_3	0.375	98.0	0.000
CO_2	C	0.280	27.0	0.700
	O	0.305	79.0	-0.350
N_2	N	0.331	36.0	-0.482
	M (COM)	0.000	0.0	0.964

Table S4: Chemical potentials (kcal/mol) of gas molecules used in the GCMC simulations at T= 363.15 K.

P (MPa)	CH_4	C_2H_6	CO_2	N_2
3	-8.56	-9.31	-9.63	-9.14
5	-8.19	-8.98	-9.36	-8.81
7	-7.96	-8.80	-9.16	-8.55
10	-7.72	-8.63	-8.95	-8.27
15	-7.43	-8.45	-8.74	-7.95
20	-7.24	-8.32	-8.62	-7.73
25	-7.09	-8.21	-8.52	-7.55
30	-6.95	-8.10	-8.44	-7.39
35	-6.83	-8.01	-8.36	-7.26
40	-6.73	-7.91	-8.29	-7.15
45	-6.63	-7.82	-8.22	-7.04
50	-6.54	-7.74	-8.16	-6.93

Table S5: Comparison n^{sat} (mmol/g) of Gurvich rule and simulation results for different fluids in rigid type IIA and type IID kerogen matrices.

Fluid	Gurvich rule				Simulation			
	IIA-1	IIA-2	IID-1	IID-2	IIA-1	IIA-2	IID-1	IID-2
CH_4	0.26	2.66	1.74	3.53	0.66	3.37	1.95	3.19
C_2H_6	0.09	1.72	1.03	2.26	0.41	3.01	1.37	2.47
CO_2	0.56	2.94	2.03	3.85	0.87	4.92	2.21	3.73
N_2	0.32	2.90	1.87	3.85	0.65	2.51	1.91	2.97

Table S6: Fitted parameters of the Tóth model.

Fluid	Kerogen model	V_{acc}	n_{max} (mmol/g)	k (1/MPa)	t	ρ_{ad} (g/cm^3)	R^2
CH_4	IIA-1	V^f_{acc}	6.53	1.75	0.24	3.42	0.95
		V^v_{acc}	2.24	0.56	0.50	1.12	0.85
	IIA-2	V^f_{acc}	8.02	0.13	0.48	0.48	0.98
		V^v_{acc}	12.39	0.10	0.39	0.34	0.97
	IID-1	V^f_{acc}	2.12	0.60	1.55	1.58	0.88
		V^v_{acc}	2.74	5.39	0.49	0.51	0.99
	IID-2	V^f_{acc}	2.77	0.28	3.22	1.45	0.88
		V^v_{acc}	3.07	0.31	1.96	0.50	0.98
C_2H_6	IIA-1	V^f_{acc}	4.65	10.00	0.22	2.00	0.89
		V^v_{acc}	1.13	99.99	0.35	5.00	0.46
	IIA-2	V^f_{acc}	3.63	0.70	0.65	0.95	0.90
		V^v_{acc}	3.52	0.43	0.84	0.60	0.99
	IID-1	V^f_{acc}	1.92	9.98	0.81	1.19	0.95
		V^v_{acc}	1.95	9.98	0.78	0.71	0.99
	IID-2	V^f_{acc}	2.80	0.44	4.59	0.84	0.99
		V^v_{acc}	2.80	0.32	49.33	0.62	0.99
CO_2	IIA-1	V^f_{acc}	8.59	81.90	0.18	10.00	0.98
		V^v_{acc}	3.45	2.75	0.43	1.64	0.98
	IIA-2	V^f_{acc}	4.42	0.47	0.92	2.97	0.91
		V^v_{acc}	6.50	0.88	0.50	1.14	0.99
	IID-1	V^f_{acc}	2.94	0.35	5.07	2.14	0.98
		V^v_{acc}	2.90	0.30	49.97	2.35	0.99
	IID-2	V^f_{acc}	5.83	199.87	0.30	1.84	0.98
		V^v_{acc}	4.32	1.22	1.19	1.34	0.99
N_2	IIA-1	V^f_{acc}	10.00	0.01	0.39	2.00	0.95
		V^v_{acc}	10.00	0.05	0.27	2.00	0.93
	IIA-2	V^f_{acc}	4.84	0.25	0.33	2.00	0.93
		V^v_{acc}	1.87	0.19	0.68	2.00	0.98
	IID-1	V^f_{acc}	1.95	1.20	0.65	4.47	0.98
		V^v_{acc}	2.09	1.40	0.58	1.34	0.89
	IID-2	V^f_{acc}	2.30	0.67	0.77	3.73	0.93
		V^v_{acc}	3.95	1.16	0.42	0.91	0.91

References

(1) Potoff, J. J.; Siepmann, J. I. Vapor–liquid equilibria of mixtures containing alkanes, carbon dioxide, and nitrogen. *AIChE J.* **2001**, *47*, 1676–1682.

(2) Martin, M. G.; Siepmann, J. I. Transferable Potentials for Phase Equilibria. 1. United-Atom Description of n-Alkanes. *J. Phys. Chem. B* **1998**, *102*, 2569–2577.

(3) Zhang, T.; Ellis, G. S.; Ruppel, S. C.; Milliken, K.; Yang, R. Effect of organic-matter type and thermal maturity on methane adsorption in shale-gas systems. *Org. Geochem.* **2012**, *47*, 120–131.

(4) Gasparik, M.; Bertier, P.; Gensterblum, Y.; Ghanizadeh, A.; Krooss, B. M.; Littke, R. Geological controls on the methane storage capacity in organic-rich shales. *Int. J. Coal Geol.* **2014**, *123*, 34–51.

(5) Ho, T. A.; Wang, Y.; Criscenti, L. J. Chemo-mechanical coupling in kerogen gas adsorption/desorption. *Phys. Chem. Chem. Phys.* **2018**, *20*, 12390–12395.

(6) Yu, X.; Li, J.; Chen, Z.; Wu, K.; Zhang, L.; Yang, S.; Hui, G.; Yang, M. Determination of CH4, C2H6 and CO2 adsorption in shale kerogens coupling sorption-induced swelling. *Chem. Eng. J.* **2021**, *410*, 127690.

(7) Liu, B.; Babaei, S.; Kanduč, M.; Tian, S.; Bai, L.; Xu, Y.; Ostadhassan, M. Helium expansion revisited: Effects of accessible volume on excess adsorption in kerogen matrices. *Chem. Eng. J.* **2024**, *493*, 152690.
